# More than meets the naked eye: an unusual psoriatic arthritis mimicry and the important role of dermoscopic examination

**DOI:** 10.1186/s41927-021-00182-7

**Published:** 2021-04-12

**Authors:** Lim Jo Anne, Mohd Jazman Che Rahim, Wan Syamimee Wan Ghazali, Wan Aireene Wan Ahmed, Seoparjoo Azmel Mohd Isa

**Affiliations:** 1 School of Medical Sciences, Universiti Sains Malaysia Health Campus, Kubang Kerian, 16150 Kota Bharu, Kelantan Malaysia; 2 Hospital Universiti Sains Malaysia, Kubang Kerian, 16150 Kota Bharu, Kelantan Malaysia

**Keywords:** Psoriatic arthropathy, Psoriatic arthritis, psoriasis, Lichen planus, Hand osteoarthritis, Heberden nodes

## Abstract

**Background:**

Psoriatic arthritis (PsA) can manifest in various forms. This includes mimicry of other diseases. We describe an unusual mimicry of PsA.

**Case presentation:**

We report a case of a middle-aged lady who presented with severe pain and morning stiffness over the small joints of the left hand for 3 months and painless deformity of the affected joints 1 year before. She was under treatment for pruritic rash over her ankles and knees for the past 1 year as well. Physical examination revealed a fixed flexion deformity, swelling and tenderness of the left ring and little fingers’ distal interphalangeal (DIP) joints. Left hand radiograph showed sclerotic joint margin, narrowed joint space and marginal osteophytes of the affected DIP joints. Dermoscopic examination showed red- violaceous, flat-topped papules and plaques with minimal scales on both ankles; hyperpigmented scaly plaques over both knees and vertical fingernail ridges. Serum autoimmune screening and inflammatory markers were unremarkable. Left ankle skin biopsy showed features consistent of psoriasis. PsA was diagnosed. Weekly titrated oral methotrexate and topical steroid were started. The patient showed significant improvement after 1 month of treatment.

**Conclusion:**

PsA is a great mimicker. Dermoscopy is an accessible and valuable tool to assess skin lesions in greater detail. Clinicians should be aware of coexisting diseases or misdiagnosis when patients do not respond to treatment.

## Background

In Malaysia, psoriasis commonly manifests in chronic plaque form (85.1%) [1]. Psoriatic arthropathy is seen in 13.7% of reported cases. Psoriasis and psoriatic arthritis (PsA) are known to manifest in various forms. It may coexist or mimic other diseases such as chronic eczema, seborrheic dermatitis, onychomycosis, lichen planus and nodular prurigo, to name a few. PsA patients are at risk of irreversible joints damage, cardiovascular disease and death if left untreated [2]. In this report, we present an unusual manifestation of this potentially disabling and deadly disease.

## Case presentation

A 50-year-old housewife presented with severe pain, early-morning stiffness and swelling over the joints of her left ring and little fingers. The symptoms started 3 months earlier and gradually worsened. The pain was worse in the morning and not relieved with rest. Pain score using IASP Pain Scale [2] ranged from 6 to 8 with temporary improvement after analgesics (paracetamol and diclofenac). She noticed that the joints were painlessly deformed 1 year earlier and had sought medical attention. The attending general practitioners had diagnosed her as having hand osteoarthritis. There was no history of trauma, fever, hair loss or oral ulcers. She had no known prior medical illnesses. There was no family history of hand osteoarthritis or any other skin or joint diseases. There was no personal or family history of atopic eczema, asthma, allergic rhinitis and allergic conjunctivitis.

The patient also noticed multiple pruritic skin rashes over both ankles and knees for the last 1 year. According to her, the rashes were hardly scaly. She was diagnosed with lichen planus by her general practitioners. However, treatment with topical agents was to no avail.

**Fig. 1:**
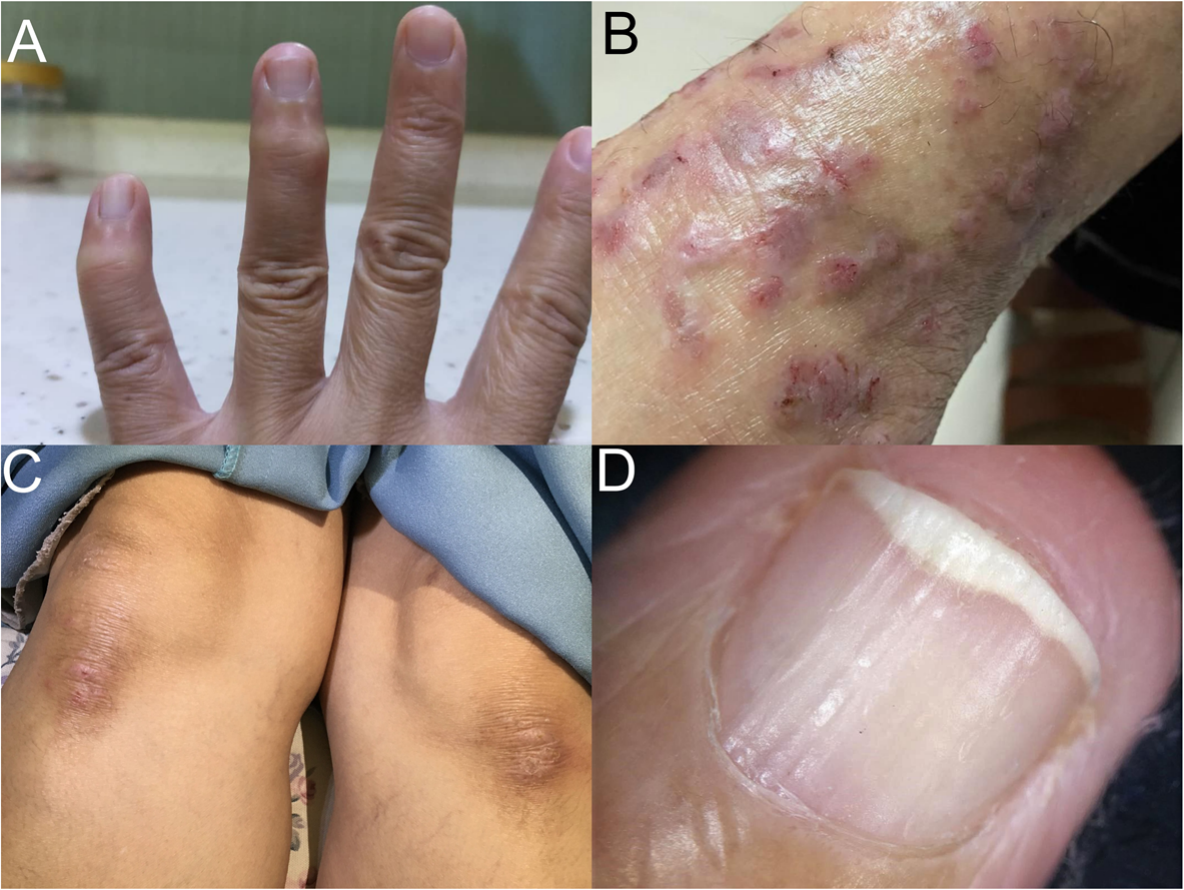
**a** Fixed flexion and nodules of DIPJ of ring and little fingers. Inward curvature of the little finger’s distal phalanx. **b** Left ankle. Multiple red-violaceous, flat-topped papules and plaques with minimal scales on the ankles and superficial excoriation. **c** Hyperpigmented scaly plaques over bilateral knee. **d** Vertical ridges on nail

**Fig. 2:**
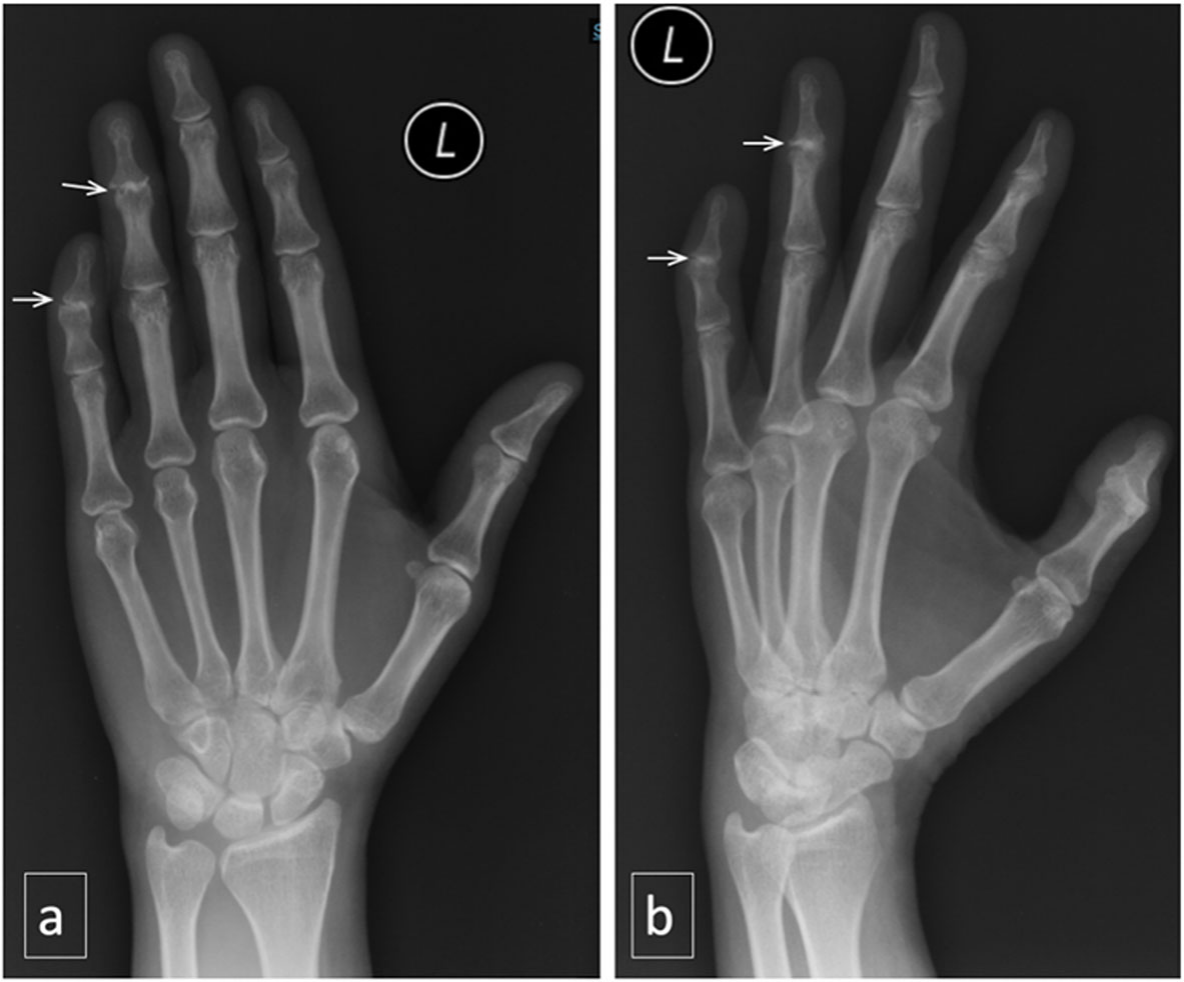
X-ray of left hand in AP (**a**) and oblique view (**b**) show abnormalities of the 4th and 5th distal interphalangeal (DIP) joints. They appear irregular with sclerotic joint margin, narrowed joint space and marginal osteophytes

Physical examination revealed deformity of the distal interphalangeal (DIP) joints of the left ring and little fingers. There was fixed flexion of both joints. The little finger’s distal phalanx was curved inward (Fig. 1a). The joints were swollen, erythematous and tender on palpation. Range of motion was also limited due to pain. Left hand radiograph showed abnormalities at the DIP joints of the ring and little fingers (Fig. 2). Both appeared irregular with sclerotic joint margin, narrowed joint space and marginal osteophytes.

Her blood investigations showed normal white cell counts (7.42 × 10^9^/L); slightly elevated erythrocyte sedimentation rate (ESR) (22 mm/hour; normal 1–20 mm/hour); antinuclear antibody (ANA), rheumatoid factor (RF) and anticyclic citrullinated protein antibody (ACCPA) were all negatives. The other blood cell counts, renal and liver profiles were normal.

There were multiple red-violaceous, flat-topped papules and plaques with minimal scales on the ankles (left more than right) and superficial excoriation (Fig. 1b). There were also hyperpigmented scaly plaques over both knees (Fig. 1c) and vertical ridges on her fingernails (Fig. 1d). Oral examination showed normal dentition with no mucosal streaks, erosion or ulcers. The rest of the skin, scalp and other systems examinations were unremarkable.

**Fig. 3:**
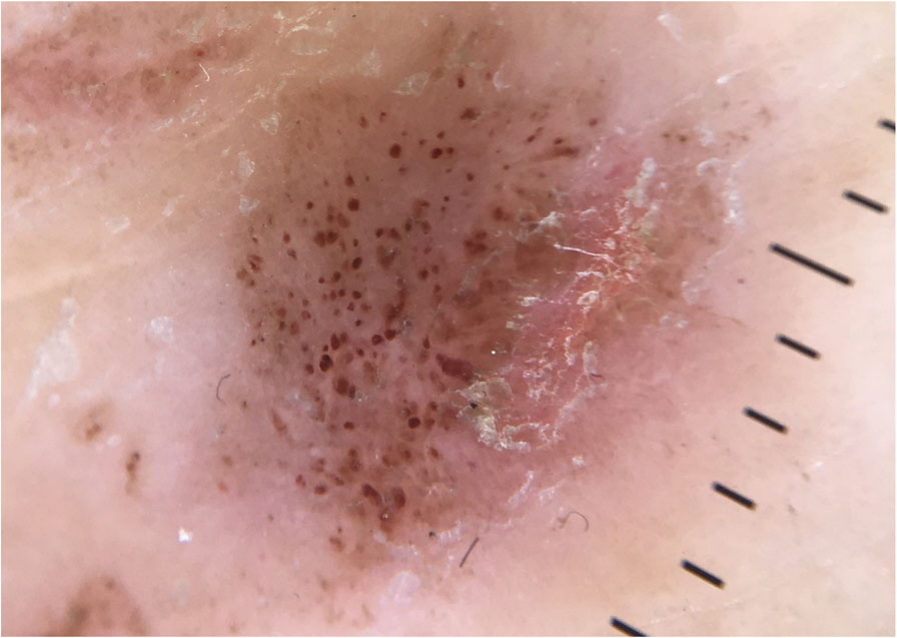
Dermoscopic examination of left ankle. Dermoscopic Auspitz sign. (polarized mode, 10x magnification)

**Fig. 4:**
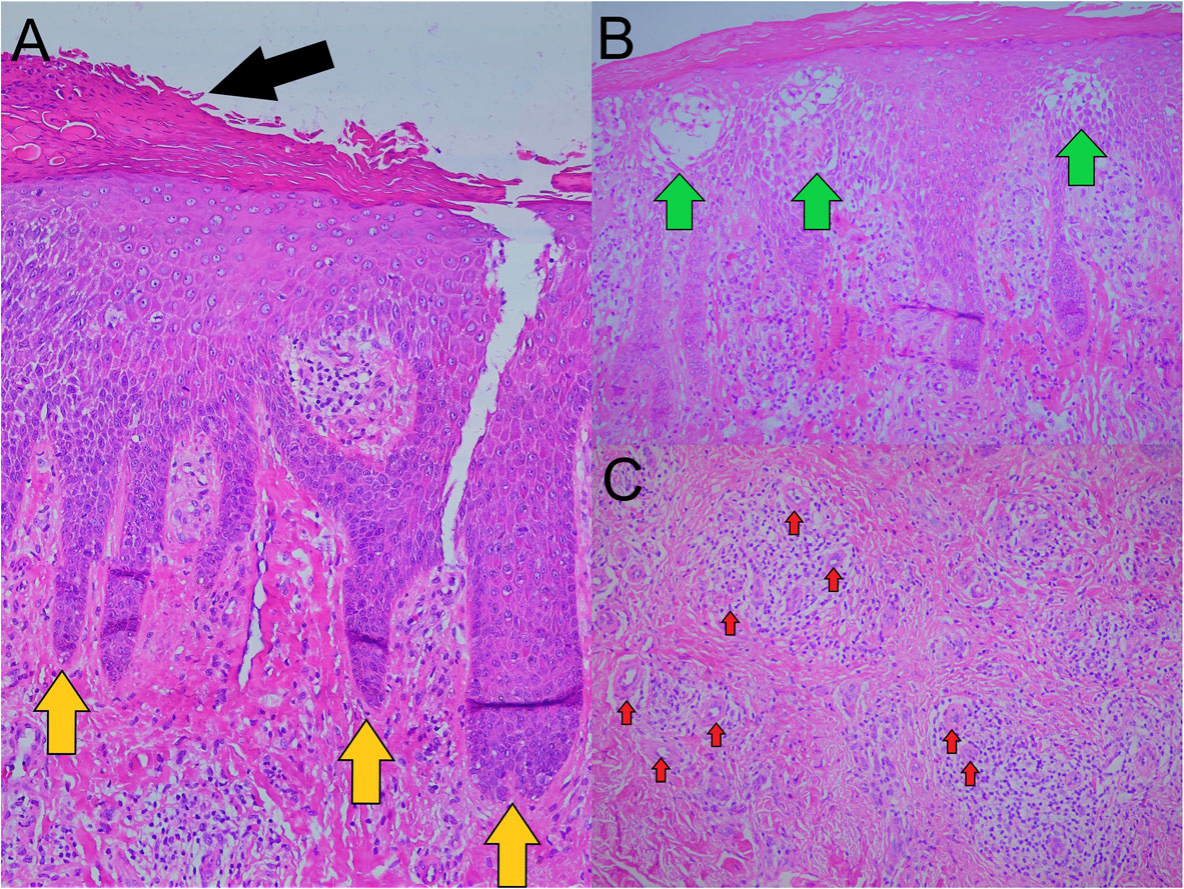
**a** Parakeratosis (black arrow) at the surface of the epidermis with elongation of the rete ridges (yellow arrow). **b** Spongiosis of the epidermal layer (green arrow). **c** Small capillaries proliferation (red arrow) with surrounding mixture of inflammatory cells, predominantly lymphocytes. (H&E stain, 40x)

Dermoscopic examination using DermLite DL4 (3Gen Inc., San Juan Capistrano, 92,675, America) revealed dermoscopic Auspitz sign over a light red background, minimal white scales with a regularly distributed dotted blood vessel (Fig. 3). Punch biopsy of the skin lesion on the left ankle (Fig. 4) showed parakeratosis at the surface of the epidermis with elongation of the rete ridges; spongiosis of the epidermal layer and small capillaries proliferation seen in the papillary dermis with surrounding mixture of inflammatory cells, predominantly lymphocytes. These findings are consistent with psoriasis.

**Fig. 5:**
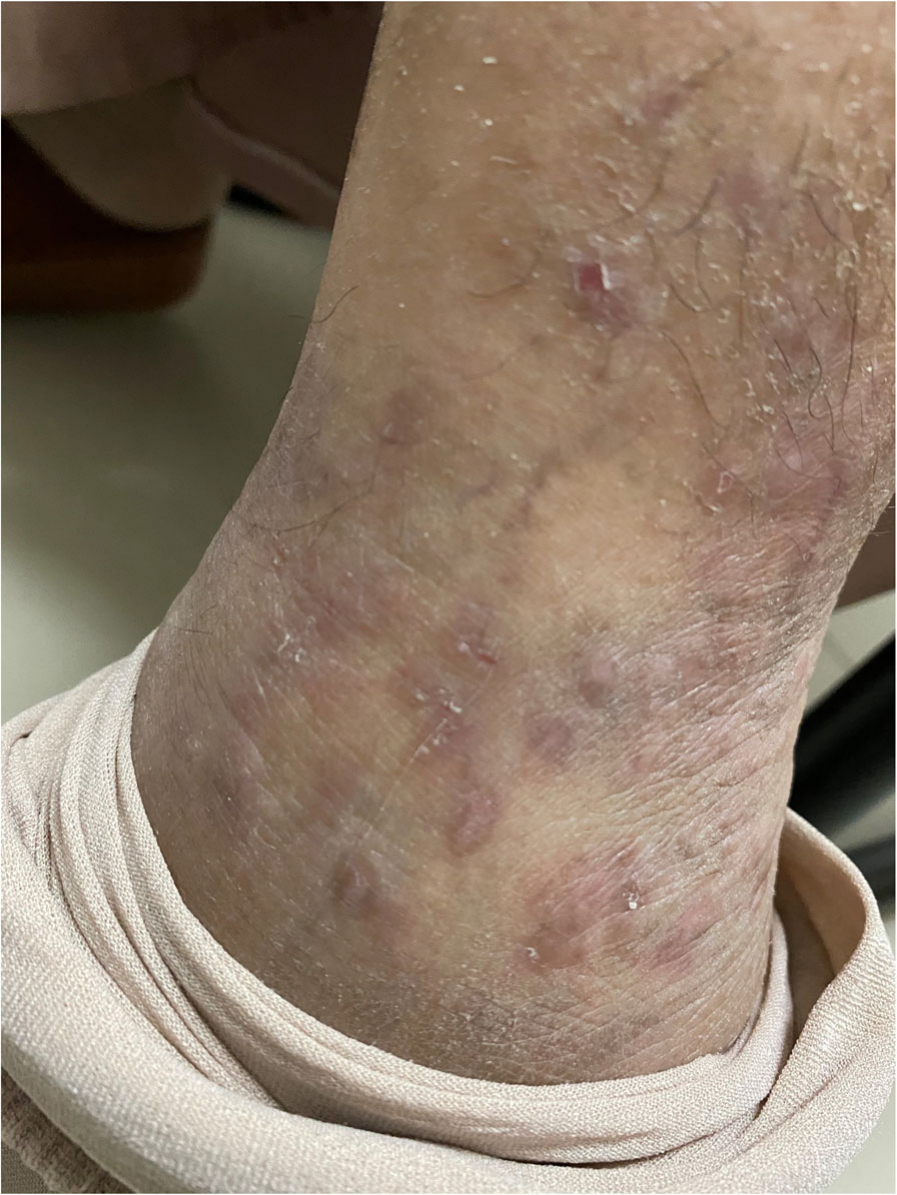
Left ankle appearance 1 month after treatment

We diagnosed her as having psoriatic arthritis (PsA). Treatment consisted of weekly oral methotrexate 7.5 mg titrated up to 15 mg with folic acid supplement and topical betamethasone valerate cream 0.5%. Her skin rash (Fig. 5) and joint pain had improved markedly after 4 weeks of treatment.

## Discussion and conclusion

Several diseases share similar clinical features with psoriasis. The commonest ones that can easily mimic psoriatic plaque over the lower legs are chronic eczema, nodular prurigo and lichen planus. Patients with chronic eczema tend to have personal or family history of atopy, asthma, allergic rhinitis, allergic conjunctivitis or potential allergen or irritant [3]. Dermoscopy of chronic eczema may present with red dots, yellow serocrust, focal dull white scales and vessels in clusters [4]. Histological findings in chronic eczema are nonspecific. These include spongiosis, intraepidermal vesicles, lymphocytic exocytosis [5].

For nodular prurigo, the itch is usually intense for days on end. The rashes are warty and crusted. Dermoscopy may reveal “white starburst pattern”. Histological examination shows increase in nerve fibre ending with a negative direct immunofluorescence staining [6].

For lichen planus, 50% of patients would have mucosa involvement and 10% will have nails involvement [7]. Wickham striae can be seen clinically or dermoscopically [8]. Histological findings include immunoglobulin deposition over the epidermal basal region from direct immunofluorescent stain.

Pruritus affects 64–97% of psoriasis patients [9]. The likely mechanism is neurogenic inflammation; a combination of abnormally expressed neuropeptides, increased innervation and peripheral opioid system dysfunction [10]. Treatment relies upon resolution of the psoriatic skin lesions, either with topical medications in most cases or systemic drugs in extensive or extra-cutaneous involvement.

Our case highlights the importance of distinguishing psoriasis from other skin disorders especially lichen planus despite obvious symptoms (severe pruritus) and clinical appearance (red-violaceous, flat-topped papules and plaques with minimal scales on ankles and knees). A dermoscopic examination of the skin lesion and nail changes may give some clue, which in our case, pointed towards psoriasis (dermoscopic Auspitz sign, light red background, white scales with a regularly distributed dotted blood vesse; nail ridges). However, other pruritic lesions may present with Auspitz sign [11].

Nail ridges are not specific for psoriasis. Psoriatic nail dystrophy can present as pitting, leukonychia, onycholysis, subungual hyperkeratosis, lines or ridges, nail plate crumbling and splinter hemorrhage [12]. Lichen planus nails involvement may present as nail plate thinning, grooves or ridges, nail darkening, nail thickening, onycholysis, pterygium and anonychia [13]. Other causes of vertical nail ridges include eczema, elderly, and nutritional deficiency [14].

Hand osteoarthritis (HOA) is common in female patients aged 40 years or older with positive family history. Joint pain usually occurs upon usage and morning/inactivity stiffness is usually mild [15]. Heberden nodes develop years after recurrent joint pain and swelling. PsA in its early course may have similar radiological appearance as HOA as seen in this case.

The occurrence of distal IPJ arthropathy; the absence of joint pain preceding the DIPJ deformity and Heberden nodes formation; the presence of resting joints pain, early morning joints stiffness, skin rash and nail changes added further credence to our suspicion of PsA. Ultimately, we confirmed the diagnosis with skin biopsy findings and clinical improvement with oral methotrexate and topical steroid. The rapidity of the hand joints deformity in our case indicates a very aggressive disease.

Psoriasis and lichen planus share some common features such as a red and scaly look. However, both have different pathology and distinct management. There are few reports of psoriasis and lichen planus coexistence [16]. There are also cases of lichen planus mimicking psoriasis [17]. However, case reports and pictures on psoriasis mimicking lichen planus are scarce.

To the best of our knowledge, this clinical mimicry has never been documented, especially among Asians. Our case also demonstrates the challenges of relying on history and gross physical examination alone for final identification of lichen planus versus psoriasis. It is important to remind clinicians of this association to avoid misdiagnosis and delay in appropriate treatment.

Non-invasive and accessible dermoscopic examination has proved to be a valuable tool in diagnosing such conditions [18]. Therefore, clinicians who frequently encounter skin diseases need to be acquainted with this important diagnostic tool. PsA may have clinical presentations that mimic other diseases or coexist with them. When response to treatment deviates from its expected course, clinicians should consider coexisting diseases and misdiagnosis.

## Data Availability

Not applicable. All data generated or analysed during this study are included in this published article.

## Abbreviations

PsA: Psoriatic arthritis
DIP: Distal interphalangeal
ESR: Erythrocyte sedimentation rate
ANA: Antinuclear antibody
RF: Rheumatoid factor
ACCPA: Anticyclic citrullinated protein antibody
HOA: Hand osteoarthritis
